# Normative misperceptions about alcohol use in the general population of drinkers: A cross-sectional survey

**DOI:** 10.1016/j.addbeh.2014.11.010

**Published:** 2015-03

**Authors:** Claire Garnett, David Crane, Robert West, Susan Michie, Jamie Brown, Adam Winstock

**Affiliations:** a Research Department of Clinical, Educational and Health Psychology, University College London, London, UK; b Cancer Research UK Health Behaviour Research Centre, University College London, London, UK; c Institute of Psychiatry, National Addiction Centre, King's College London, London, UK; d South London and Maudsley NHS Foundation Trust, King's College London, London, UK

**Keywords:** Normative misperception, Alcohol, AUDIT

## Abstract

**Introduction:**

Underestimating one's own alcohol consumption relative to others (‘normative misperception’) has been documented in some college student and heavy-alcohol using samples, and may contribute to excessive drinking. This study aimed to assess how far this phenomenon extends to alcohol users more generally in four English-speaking countries and if associations with socio-demographic and drinking variables exist.

**Methods:**

A cross-sectional online global survey (Global Drugs Survey-2012) was completed by 9820 people aged 18 + from Australia, Canada, the UK and US who had consumed alcohol in the last year. The survey included the AUDIT questionnaire (which assessed alcohol consumption, harmful drinking and alcohol dependence), socio-demographic assessment and a question assessing beliefs about how one's drinking compares with others. Associations were analysed by linear regression models.

**Results:**

Underestimation of own alcohol use relative to others occurred in 46.9% (95% CI: 45.9%, 47.9%) of respondents. 25.4% of participants at risk of alcohol dependence and 36.6% of harmful alcohol users believed their drinking to be average or less. Underestimation was more likely among those who were: younger (16–24; *p* < 0.003), male (*p* < 0.001), from the UK (versus US; *p* < 0.001), less well educated (*p* = 0.003), white (*p* = 0.035), and unemployed (versus employed; *p* < 0.001).

**Conclusions:**

Underestimating one's own alcohol consumption relative to other drinkers is common in Australia, Canada, the UK and US, with a substantial minority of harmful drinkers believing their consumption to be at or below average. This normative misperception is greater in those who are younger, male, less well educated, unemployed, white, from the UK and high-risk drinkers.

## 1 Introduction

‘Normative misperception’ about alcohol use refers to the underestimation of one's own alcohol consumption relative to others. There is a reason to believe that normative misperception may play a role in excessive alcohol consumption as studies have found that providing normative feedback can reduce subsequent alcohol use (Collins, Carey, & Sliwinski, 2002; Cunningham, Neighbors, Wild, & Humphreys, 2012; Cunningham, Wild, Bondy, & Lin, 2001; Kypri & Langley, 2003; Neighbors, Larimer, & Lewis, 2004; Wild, 2002). It is important to establish how widespread normative misperceptions are and what factors may underlie them. This paper addresses those issues.

Research on normative misperception has been limited to college and university students (Baer, Stacy, & Larimer, 1991; Kypri & Langley, 2003; Neal & Carey, 2004; Neighbors, Dillard, Lewis, Bergstrom, & Neil, 2006; Neighbors et al., 2004) or heavy drinking samples (Cunningham et al., 2001, 2012). It has been found that these groups tend to underestimate their alcohol consumption relative to other people.

There is very little research on correlates of normative misperception. Two studies (Larimer, Irvine, Kilmer, & Marlatt, 1996; Prentice & Miller, 1993) have found that women perceived larger differences between their own and others' drinking behaviour but another study (Read, Wood, Davidoff, McLacken, & Campbell, 2002) found no effect of gender on perceived norms for quantity or frequency of alcohol consumption. To our knowledge, no other correlates of normative misperception have been investigated.

This study aimed to assess the prevalence of this phenomenon in a more diverse sample spanning four English-speaking countries, and to examine associations between the phenomenon and socio-demographic and drinking variables.

This study addressed the following research questions:

1. What is the prevalence of normative misperceptions about alcohol use in the general population of alcohol users from the UK, US, Australia, and Canada?
2. To what extent are normative misperceptions about alcohol use associated with a range of socio-demographic and drinking variables?

## 2 Methods

### 2.1 Study design

This was an anonymous cross-sectional online survey conducted in 116 countries (Global Drugs Survey (GDS) -2012). Sample sizes for four English-speaking countries were sufficiently large to provide useful data and these formed the basis for the study. The GDS has been developed by an expert advisory group and an academic network, and captures information to monitor the use of drugs and identify emerging trends in drug use (McCambridge, Mitcheson, Winstock, & Hunt, 2005; Winstock & Barratt, 2013a,b; Winstock, Griffiths, & Stewart, 2001; Winstock et al., 2011). Participants were recruited using a purposive sampling strategy (McCambridge et al., 2005).

### 2.2 Participants

This study draws on GDS data obtained from Australia, Canada, the UK or the US (*n* = 12,309). Participants who were 18 years old or over, had answered “yes” to whether they had used alcohol within the last 12 months and had no missing data for any of the variables were included in this study. This resulted in 9820 participants whose demographic characteristics are shown in Table 1. The majority of participants were aged between 16 and 24, male, white, from the UK, had post-16 qualifications, and were employed.

### 2.3 Measures

Alcohol use and problems associated with it were assessed using the Alcohol Use Disorders Identification Test (AUDIT) (Babor, Higgins, Saunders, & Monteiro, 2001). The full 10-item AUDIT questionnaire assesses alcohol consumption, harmful drinking and alcohol dependence. The possible scores range from 0 to 40 and are categorised into four risk zones: Zone 1 (0–7) refers to low-risk drinking or abstinence; Zone 2 (8–15) refers to hazardous drinking; Zone 3 (16–19) refers to harmful drinking; and Zone 4 (20–40) identifies those who are at risk of alcohol dependence and warrant further assessment and investigation (Babor et al., 2001). The AUDIT alcohol consumption (AUDIT-C) questionnaire consists of the first three-items of the full AUDIT questionnaire.

Normative perceptions about alcohol use were assessed by the question: “How do you think your use of alcohol compares to other people who have used that substance recently?” Participants selected one of nine categories and ‘Don't know’: 1 = Lowest 10%, 2 = Very low, 3 = Low, 4 = Low–average, 5 = Average (middle 20%), 6 = High average, 7 = High, 8 = Very high, and 9 = Top 10%.

Socio-demographic information on age, gender, ethnicity, country of origin, employment status, and highest qualification level attained was collected.

### 2.4 Procedure

The GDS (https://www.globaldrugsurvey.com) was actively promoted as an anonymous, online survey about drug use through social networking sites (e.g. Twitter, Facebook) for five weeks from November 16th 2011. The promotions invited people to take part in a study investigating drug use and related attitudes and included a link to the study hosted on the GDS website. Those interested in participating after reading the study information were asked for informed consent prior to submission of their completed questionnaire. Respondents were offered no incentive for participation. The average time for completion was approximately 35 min.

Ethical approval was granted by the Joint South London and Maudsley and Institute of Psychiatry NHS Research Ethics Committee (reference number 141/02).

### 2.5 Analysis

The AUDIT-C was used to calculate the normative misperception score as it focuses on alcohol consumption. The middle two deciles of AUDIT-C scores were combined into one category so that the AUDIT-C score deciles could be directly compared with the nine-item scale of normative misperception which was anchored on the lowest 10%, the middle 20% and the highest 10% (see above). This yielded an AUDIT ‘position’ from 1 to 9 (1 = 0–10%, 2 = 10–20%, 3 = 20–30%, 4 = 30–40%, 5 = 40–60%, 6 = 60–70%, 7 = 70–80%, 8 = 80–90%, 9 = 90–100%). The ‘normative misperception score’ was calculated as the difference between each participant's actual AUDIT-C position and their rating, and could range from − 8 to + 8. A positive score indicates that an individual underestimated their alcohol use compared with others whilst a negative score corresponds to an overestimation. The magnitude of the normative misperception score corresponds to the extent of discrepancy between the individual's actual and perceived position in the AUDIT-C distribution. This method operationalises normative misperceptions for the purposes of assessing associated factors and the magnitude of the normative misperceptions.

The prevalence of normative misperception for different AUDIT risk zones was assessed through cross tabulation. A series of simple linear regressions were used to investigate the univariate association between the normative misperception score and the socio-demographic and drinking variables. A multiple regression model, including all the socio-demographic and drinking variables, was used to investigate which of the factors had a unique association with the normative misperception score.

## 3 Results

### 3.1 Prevalence of normative misperception

The mean normative misperception score was 0.20 (SD = 1.85) which was significantly greater than 0 (t_(9819)_ = 10.443, *p* < 0.001). This means that overall there was a small but significant tendency to underestimate one's alcohol consumption relative to others. Nearly half of the sample (46.9%, 95% CI = 45.9%, 47.9%) underestimated the proportion of other people who consume less alcohol than them whilst 38.6% (95% CI = 37.6%, 39.5%) overestimated it and 14.5% (95% CI = 13.8%, 15.2%) were accurate in their perception.

### 3.2 Univariate associations with socio-demographic variables

Country of origin, age, gender, ethnicity, employment status and qualification level were all associated with normative misperceptions (see Table 2). Respondents from the UK had significantly greater mean normative misperception scores compared with those from Australia, Canada or the US. Larger normative misperceptions (indicating an underestimation of own alcohol consumption relative to others) were more likely in participants who were younger (16–24), male, categorised themselves as ‘white’ compared with all other ethnicities, unemployed and whose highest level of qualification attained was pre-16.

### 3.3 Associations with AUDIT risk zone

AUDIT risk zone was associated with normative misperception with lowest risk drinkers having the lowest mean misperception score (see Table 2). The mean normative misperception scores for those participants who were classified as hazardous alcohol users (AUDIT risk zone 2), harmful alcohol users (risk zone 3) or at risk of alcohol dependence (risk zone 4) were significantly greater than 0 (hazardous: mean = 0.5, SD = 1.73, t_(4257)_ = 20.17, *p* < 0.001; harmful: mean = 1.1, SD = 1.74, t_(1060)_ = 20.64, *p* < 0.001; at risk of dependence: mean = 1.4, SD = 1.69, t_(885)_ = 24.18, *p* < 0.001) whereas low-risk drinkers had a normative misperception score of significantly less than 0 (mean = − 0.8, SD = 1.60, t_(3614)_ = − 28.67, *p* < 0.001). The tendency for higher risks to have higher mean normative misperceptions was also illustrated by an examination of the data categorically: 25.4% of alcohol users at risk of alcohol dependence and 36.6% of harmful alcohol users believed their alcohol use to be average or less than average.

### 3.4 Fully adjusted model

In a fully adjusted model, normative misperceptions were more likely among participants who were younger, male, from the UK compared with the US, without post-16 qualifications, white, and unemployed compared with employed (see Table 2). Those with lower levels of alcohol use (AUDIT risk zone 1) had significantly lower misperception scores than those who used alcohol more heavily (AUDIT risk zones 2, 3 & 4, ps < 0.001).

## 4 Discussion

In a large sample of alcohol users from four English-speaking countries, there was evidence of a small but significant tendency to underestimate one's alcohol consumption relative to others. This tendency was greatest amongst those who were: young (16–24), male, from the UK compared with the US, without post-16 qualifications, classifying themselves as white, and unemployed compared with employed. It was greater among those with higher AUDIT scores; 25.4% of the drinkers at risk of alcohol dependence and 36.6% of harmful drinkers considered that their consumption was average or below.

The findings confirm and extend previous research on students and heavy drinkers but show that the phenomenon is broadly restricted to heavier drinkers and light drinkers typically overestimate their drinking relative to others. If one's judgement about how one's drinking compares with others has an influence on how much one drinks, this suggests that it would be important, when feeding back information, only to highlight misperceptions when they go in one direction: namely believing one drinks the same as or less than others. A review of interventions correcting normative misperceptions did conclude that it could lead to a reduction in alcohol misuse (Moreira, Smith, & Foxcroft, 2010) but it is not clear whether the interventions worked through the intended mechanism. Future research should examine this moderation and whether there is more impact for interventions when they are targeted by socio-demographic and drinking characteristics associated with normative misperception.

One study limitation was that the distribution of AUDIT scores were derived from the GDS-2012 sample and is not representative of the general population (Friedman, 2006). Insofar as the GDS-sample was biased towards a heavy drinking sample, the results of this study are likely to be an overestimate of the overall population prevalence because the consumption comparator (from which the misperceptions were calculated) would be higher than from the general population. Secondly, the nine-point scale of AUDIT-C scores was created using all four countries (Australia, Canada, United Kingdom, and United States), though people may have answered the comparison question in relation to people in their own country. However, a sensitivity analysis using only the large UK or US sub-samples showed similar patterns of results compared with the analysis for all four countries. A third limitation relates to the way the misperception score was derived. There are many different ways in which it could have been done. This method was chosen as being the best compromise between precision in terms of intended meaning and using language that respondents could understand. We considered it best to anchor the extremes and the middle with deciles and use linguistic terms for the other response options. It is possible that different choices would result in different estimates. However, we would argue that the key findings would remain.

In conclusion, normative misperceptions about alcohol use are common in the population of alcohol users in four English-speaking countries (UK, US, Australia and Canada). The UK shows this to the greatest extent. It is common for harmful alcohol users and those at risk of dependence to believe that they drink at or less than average, and normative misperceptions tend to be greater in those who are younger, male, less well educated, unemployed and white.

## Role of funding sources

The Global Drugs Survey is funded by AW. CG is funded by the UK Centre for Tobacco and Alcohol Studies (UKCTAS) – 513533 (CG’s studentship); MR/L501475/1 (grant ref for UKCTAS). DC is funded by the National Institute for Health Research (NIHR)'s School for Public Health Research (SPHR) – 513997 (DC’s studentship). The views expressed are those of the authors and not necessarily those of the NHS, the National Institute for Health Research or the Department of Health. SPHR is a partnership between the Universities of Sheffield; Bristol; Cambridge; Exeter; UCL; The London School for Hygiene and Tropical Medicine; the LiLaC collaboration between the Universities of Liverpool and Lancaster and Fuse; The Centre for Translational Research in Public Health, a collaboration between Newcastle, Durham, Northumbria, Sunderland and Teesside Universities. JB's post is funded by a fellowship from the UK Society for the Study of Addiction – JB’s fellowship: 508045; RW is funded by Cancer Research UK - 511357. The research team is part of the UK Centre for Tobacco and Alcohol Studies, a UKCRC Public Health Research Centre of Excellence. Funding from the Medical Research Council, British Heart Foundation, Cancer Research UK, Economic and Social Research Council and the National Institute for Health Research under the auspices of the UK Clinical Research Collaboration, is gratefully acknowledged.

## Contributions

The study was conceived by all authors. The survey was designed by AW. Data was collected by AW. Data were analysed by CG, RW & JB. The manuscript was prepared and amended by CG. All authors contributed to and have approved the final manuscript.

## Conflict of interest

AW is founder and director of Global Drugs Survey. JB has received an unrestricted research grant from Pfizer related to the surveillance of smoking cessation trends. RW has received research funding and undertaken consultancy for companies that manufacture smoking cessation medications. CG, DC and SM have no competing interests.

## Figures and Tables

**Table 1:** Demographic characteristics.

Variable	*n* = 9820
Mean (SD) AUDIT score	10.5 (6.2)
AUDIT risk zone (%)	
1	36.8
2	43.4
3	10.8
4	9.0
Age (%)	
16–24	44.9
25–34	36.8
35–44	12.2
45–54	4.4
55 +	1.6
Gender (% male)	68.7
Ethnicity (% white)	92.0
Country of origin (%)	
Australia	3.1
Canada	6.5
UK	63.9
US	26.5
Qualifications (% post-16)	95.8
Employment status (%)	
Employed	49.3
Student	27.7
Unemployed	23.0

**Table 2:** The effect of socio-demographic variables and AUDIT risk zone on normative misperception score.

				Unadjusted simple linear regression				Adjusted multiple regression (with all variables as covariates)			
		*N*	Mean normative misperception score (SD)	*B*	95% CI for *B*		*p*	*B*	95% CI for *B*		*p*
					Lower bound	Upper bound			Lower bound	Upper bound	
Country of origin	United Kingdom^a^	6273	0.4 (1.78)								
	Australia	306	0.2 (1.95)	− 0.25	− 0.46	− 0.04	0.021	0.03	− 0.16	0.22	0.779
	Canada	641	0.1 (1.92)	− 0.36	− 0.50	− 0.21	< 0.001	− 0.02	− 0.16	0.11	0.753
	United States	2600	− 0.3 (1.90)	− 0.70	− 0.78	− 0.61	< 0.001	− 0.29	− 0.37	− 0.21	< 0.001
AUDIT risk zone (AUDIT score)	1 (0–7)^a^	3615	− 0.8 (1.60)								
	2 (8–15)	4258	0.5 (1.73)	1.40	1.33	1.47	< 0.001	1.29	1.21	1.36	< 0.001
	3 (16–19)	1061	1.1 (1.74)	2.04	1.92	2.16	< 0.001	1.90	1.77	2.02	< 0.001
	4 (20–40)	886	1.4 (1.69)	2.19	2.03	2.34	< 0.001	2.00	1.85	2.16	< 0.001
Age/years	16–24^a^	4407	0.5 (1.88)								
	25–34	3615	0.0 (1.80)	− 0.44	− 0.52	− 0.36	< 0.001	− 0.28	− 0.36	− 0.20	< 0.001
	35–44	1201	0.0 (1.79)	− 0.50	− 0.62	− 0.38	< 0.001	− 0.22	− 0.34	− 0.11	< 0.001
	45–54	436	− 0.2 (1.77)	− 0.67	− 0.86	− 0.49	< 0.001	− 0.26	− 0.43	− 0.09	0.003
	55 +	161	− 0.6 (1.71)	− 1.04	− 1.33	− 0.76	< 0.001	− 0.47	− 0.73	− 0.21	< 0.001
Gender				0.47	0.39	0.55	< 0.001	0.34	0.27	0.41	< 0.001
	Male	6750	0.3 (1.84)								
	Female	3070	− 0.1 (1.84)								
Qualification level				− 0.44	− 0.62	− 0.26	< 0.001	− 0.25	− 0.41	− 0.08	0.003
	Pre-16	412	0.6 (1.91)								
	Post-16	9408	0.2 (1.85)								
Employment status	Unemployed^a^	2256	0.4 (1.91)								
	Student	2718	0.3 (1.87)	− 0.13	− 0.23	− 0.02	0.018	− 0.09	− 0.18	0.00	0.056
	Employed	4846	0.1 (1.80)	− 0.36	− 0.45	− 0.26	< 0.001	− 0.20	− 0.29	− 0.11	< 0.001
Ethnicity				− 0.27	− 0.40	− 0.13	< 0.001	− 0.13	− 0.25	− 0.01	0.035
	White	9037	0.2 (1.85)								
	Non-white	783	− 0.1 (1.82)								

a Reference group for the categorical variable.
